# Supplementary material for: The interactome of intact mitochondria by cross-linking mass spectrometry provides evidence for coexisting respiratory supercomplexes
Source: Mol Cell Proteomics. 2017 Dec 8;17(2):216–32. doi: 10.1074/mcp.RA117.000470 (PMC5795388; doi:10.1074/mcp.RA117.000470)
Supplement: Supplemental Data [file supp_RA117.000470_133922_0_supp_23978_fzffwf.zip › spectra_annotation/mito_spectra_annotation/49-1-87-1-65-1.pdf]

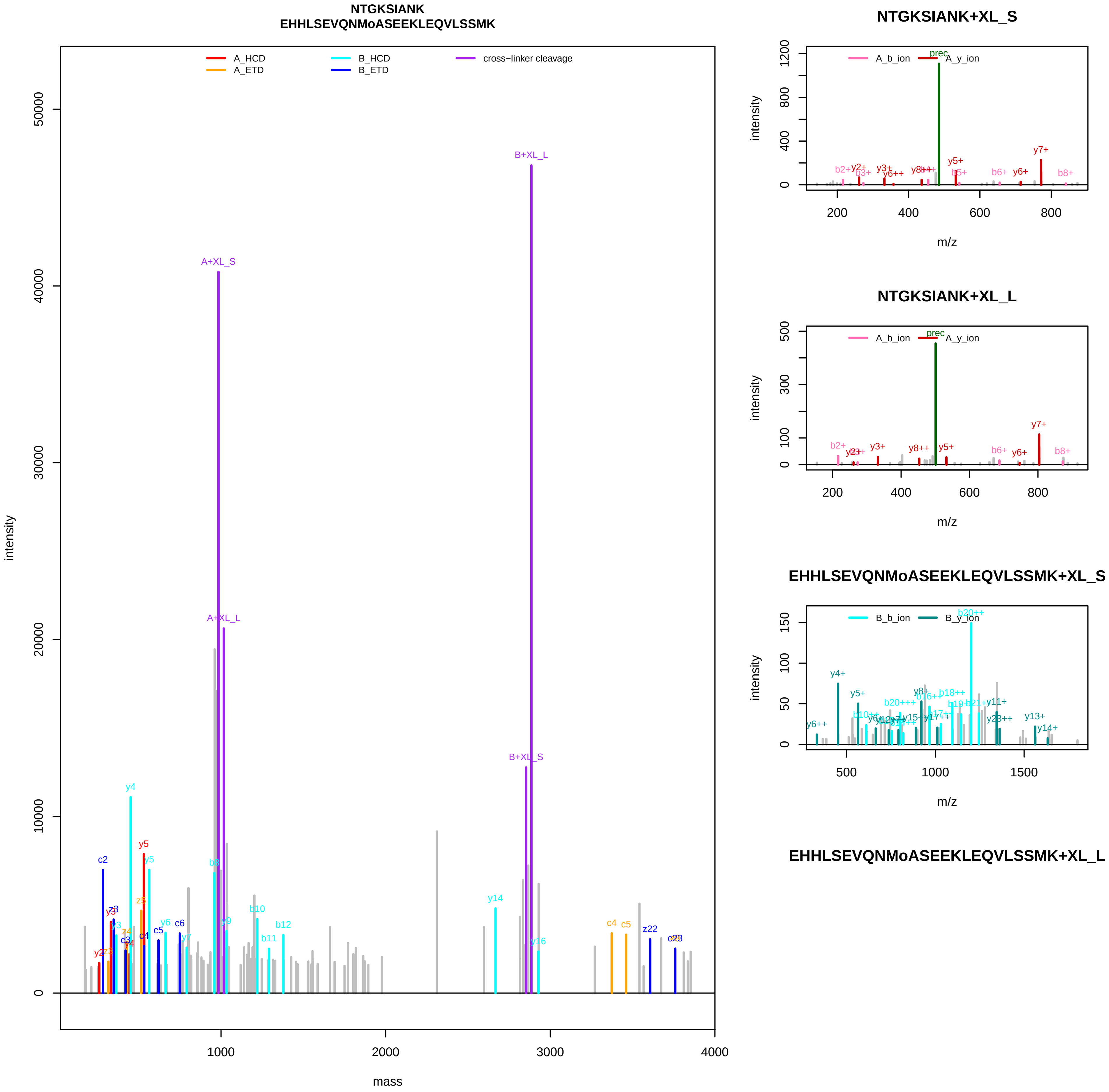
NTGKSIANK
EHHLSEVQNMoASEEKLEQVLSSMK
A_HCD
A_ETD
B_HCD
B_ETD
cross−linker cleavage
intensity
mass
0
10000
20000
30000
40000
50000
1000
2000
3000
4000
A+XL_S
A+XL_L
B+XL_L
B+XL_S
y4
y5
c2
y14
b10
b12
b11
c4
c5
z22
c23
NTGKSIANK+XL_S
NTGKSIANK+XL_L
EHHLSEVQNMoASEEKLEQVLSSMK+XL_S
EHHLSEVQNMoASEEKLEQVLSSMK+XL_L
A_b_ion
A_y_ion
B_b_ion
B_y_ion
prec
m/z
y7+
y5+
y6+
b8+
b6+
y4+
y5+
y8+
y11+
y13+
y14+
y6++
b20++
